# Metformin: On Ongoing Journey across Diabetes, Cancer Therapy and Prevention

**DOI:** 10.3390/metabo3041051

**Published:** 2013-11-07

**Authors:** Claudio Pulito, Toran Sanli, Punam Rana, Paola Muti, Giovanni Blandino, Sabrina Strano

**Affiliations:** 1 Molecular Chemoprevention Group, Molecular Medicine Area, Regina Elena National Institute, Rome 00144, Italy; E-Mail: c.pulito@ifo.it; 2 Department of Oncology, Juravinski Cancer Center, McMaster University, Hamilton, ON L8V 5C2, Canada; E-Mails: sanlit@mcmaster.ca (T.S.); punam.rana@gmail.com (P.R.); muti@mcmaster.ca (P.M.); 3 Translational Oncogenomics Unit-ROC, Molecular Medicine Area, Regina Elena National Institute, Rome 00144, Italy; E-Mail: blandino@ifo.it

**Keywords:** diabetes, cancer, microRNA, therapy, chemoprevention, metabolism, meta analysis

## Abstract

Cancer metabolism is the focus of intense research, which witnesses its key role in human tumors. Diabetic patients treated with metformin exhibit a reduced incidence of cancer and cancer-related mortality. This highlights the possibility that the tackling of metabolic alterations might also hold promising value for treating cancer patients. Here, we review the emerging role of metformin as a paradigmatic example of an old drug used worldwide to treat patients with type II diabetes which to date is gaining strong *in vitro* and *in vivo* anticancer activities to be included in clinical trials. Metformin is also becoming the focus of intense basic and clinical research on chemoprevention, thus suggesting that metabolic alteration is an early lesion along cancer transformation. Metabolic reprogramming might be a very efficient prevention strategy with a profound impact on public health worldwide.

## 1. Introduction: The Evolution of Metformin

Currently, the anti-hyperglycemic agent metformin (dimethylbiguanide) is the front-line therapy for patients with type II diabetes (T2D) worldwide. Its medical history dates back to the use of *Galega officinalis* (the French lilac), which was utilized in Chinese medicine and also in medieval Europe to treat halitosis and polyurea [1,2]. Later, this plant was also described to treat symptoms of diabetes up until the early 1930s in France [3]. Research in the late 1800s found that *Galega officinalis* was rich in guanidine, which had hypoglycemic proprieties in animals that may explain the plants anti-diabetic action [4]. However, the clinical use of guanidine was found to be toxic, but an isoprenyl derivative, known as galegine, had fewer side-effects and was used for the treatment of diabetes in humans in the 1920s [5]. At around the same time, dimethylbiguanide (now known as metformin) was also synthesized and effectively lowered blood glucose levels *in vivo* [6], but its clinical application in treating diabetes was hindered by the discovery of insulin during the same decade. Not until the 1950s was metformin, as well as the more potent biguanide derivatives phenformin and buformin, used clinically for the treatment T2D [7]. Initially, the latter drugs were more widely used, however phenformin and buformin were correlated with life-threatening lactic acidosis which led to their discontinuation in the 1970s [8]. Meanwhile, metformin use began to thrive due to its high therapeutic index.

Clinically it has been shown that metformin works to suppress hepatic gluconeogenesis, thereby lowering blood glucose levels in patients with poorly managed T2D [9]. It should be noted though, that the molecular mechanisms by which metformin achieves these effects are still largely debated. However, a prevailing premise is that due to its positive charge, metformin accumulates within the cellular mitochondrial matrix and inhibits complex I of the mitochondrial respiratory chain (as does phenformin), which results in a backlog of ATP production [10,11]. This in turn, leads to the activation of the energy sensing enzyme AMP-activated protein kinase (AMPK), which inhibits energy consuming processes and switches cellular metabolism towards energy production to restore energy homeostasis [12]. Indeed, metformin-mediated AMPK activation results in modulation of downstream targets that enhance glucose uptake into skeletal muscle [13] and inhibit genes that regulate hepatic gluconeogenesis [14], which may explain the abovementioned clinical observations of this drug.

Due to the safety profile of metformin, this agent has gone onto numerous clinical trials for the management of other disease pathologies, including polycystic ovarian syndrome [15,16] and metabolic syndrome [17] with some success. More recently, there has been a great deal of interest in the ability of metformin in cancer chemoprevention and therapy [18]. An initial epidemiological report conducted by Evans *et al.* [19] gained the attention of the oncology field when they found that diabetic patients taking metformin, as compared to other patients treated with other hypoglycemic therapies, had a significant reduction in cancer risk. These results sparked widespread metformin research, ranging from the mechanistic studies to determine its anti-proliferative effect in cancer cells, to clinical trials in non-diabetic patients with various malignancies [20,21].

An additional benefit for metformin use in oncology is that its known to modulate energy metabolism, which is a topic that is re-emerging in the cancer field. For instance, cancer cells are often more metabolically active than surrounding non-malignant tissue. As a consequence of this phenotype, any opposition to glucose utilization by low-energy mimetics such as metformin may inhibit tumor proliferation. In fact, recent studies have indicated that tumors carrying mutations in metabolic stress regulators such as LKB1 and p53 undergo substantial apoptosis when treated with biguanides [22,23,24].

Herein, we review the metabolomic effects of metformin and highlight its possibilities and pitfalls for cancer chemoprevention and treatment. We begin by identifying the metabolic profile of cancer cells and outline the molecular mechanisms that contribute to altered energy metabolism. We then provide insight into metformin effects on these metabolic pathways and its role in the inhibition of tumor growth and proliferation, particularly at the level of microRNA (miRNA) signaling. Finally, we summarize the past and current preclinical and clinical trials that support the use of metformin for combination cancer therapy and chemoprevention.

## 2. Metabolism and Cancer

Tumorigenesis is a multistep process and reflects genetic alterations that drive cancer progression. A decade ago six essential hallmarks that represent the essential alterations in cancer cell physiology: self-sufficiency in growth signals, tissue invasion and metastasis, evasion of apoptosis, sustained angiogenesis, limitless replicative potential, insensitivity to anti-growth signals were described [25]. Currently, a revival in research on tumor metabolism also suggests that there is another important hallmark that characterized cancer cells: altered cell bioenergetic pathways [26].

Tumor cells often display alterations in their energy metabolism to support rapid growth. For example, unlike normal cells, tumors have enhanced rates of glucose uptake for glycolytic ATP generation; a truncated tricarboxylic acid (TCA) cycle utilized to produced acetyl-CoA used for the synthesis of fatty acids, cholesterol and isoprenoids; and a increase in protein and DNA synthesis due to their high rate of metabolism [27]. This physiological process was first observed by Otto Warburg almost a century ago, where he observed that cancer cells preferentially utilize glucose through aerobic glycolysis even in an oxygen rich environment [28]. This mechanism of rapid glucose utilization through glycolysis for energy and macromolecule synthesis was later termed the Warburg effect.

The Warburg effect is modulated by the activity of well-known oncogenes and tumor suppressors that are deregulated during the early stages of carcinogenesis.

**Figure 1:**
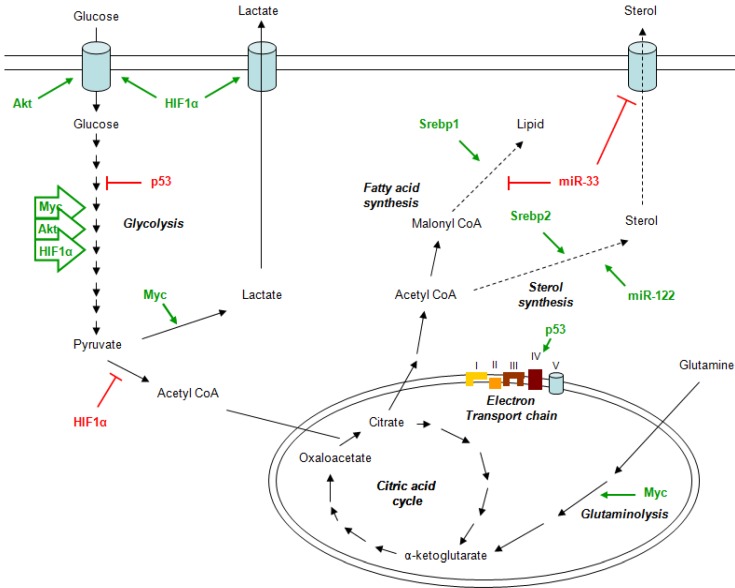
The role of oncogenes, tumor suppressors and microRNAs in cancer metabolism.

For example, the hypoxia-inducible factor 1 (HIF-1) drives the increase of glycolysis by modulating the transcription of the cell-surface transporter of glucose GLUT1 [29] and inhibits mitochondrial respiration through the activation of the pyruvate dehydrogenase kinase (PDK) [30,31] that in turn induces a decrease in Acetyl-CoA levels through the inhibition of the mitochondrial pyruvate dehydrogenase (PDH) complex (Figure 1). Independently from HIF-1, during normoxia, high levels of Akt can also increase the glycolytic flux. This event is sufficient to trigger aerobic glycolysis due to the increase in glucose uptake [32] and to the activation of hesokinase 2 (HK2) [33].

An important oncogene involved in metabolism is c-Myc, which is able to enhance directly the glycolytic pathway (Figure 1) modulating the expression of several genes involved in this process and indirectly regulate other transcription factor. In particular, it is well known that lactate dehydrogenase A (LDHA), the enzyme that converts pyruvate to lactate, is transcriptionally controlled by c-Myc [34]. In addition, c-Myc promoted mitochondrial biogenesis thereby increasing mitochondrial function [34,35]. The c-Myc-control of the mitochondrial function is important to generate substrates for macromolecular biosynthesis that guarantee a rapid cells proliferation, a typical hallmark of cancer cells and the Warburg effect. The paradoxical production of lactate together with the activation of the TCA cycle could be explained by another metabolic process mediated by c-Myc: the catabolism of glutamine [36,37] (Figure 1).

Conversely, the tumor suppressor p53 is able to suppress glycolysis by modulating the expression of two important genes, the glycolytic inhibitory protein TP53-induced glycolysis and apoptosis regulator (TIGAR) [38,39], and cytochrome c oxidase 2 subunit (SCO2) [39]. The latter stimulates mitocondrial oxidative phosphorylation through complex IV of the electron transport chain [39] (Figure 1).

Furthermore, the overexpression of genes involved in fatty acid synthesis, such as the fatty acid synthase (FASN), acetyl-CoA carboxylase alpha (ACACA) and, the ATP citrate lyase (ACLY) can induce the development and progression of cancer [40]. Overexpression of FASN, in fact, is a predictor of recurrence in stage I breast carcinoma [41], lung carcinoma [42], endometrial carcinoma [43] patients and correlate with a worst prognosis in breast carcinoma [44] and ovarian carcinoma [45] patients. In addition, ACACA is also known to be upregulated and support the proliferation of many cancers including breast, colon, lung, and liver [46].

Emerging studies have identified that microRNAs may also play a role in regulating metabolic processes. Indeed, glucose metabolism seems to be regulated by microRNAs through the modulation of the insulin production and secretion.

MicroRNAs are short and stable non coding RNAs, 18–25 nucleotides long, that modulate the translation of mRNA of coding genes by binding their 3′-untraslated regions [47]. This generally leads to the degradation of target mRNA, thereby effectively reducing the levels of the gene products. Interestingly, one microRNA can bind a plethora of mRNAs that in turn can be bonded by several microRNAs. For these reasons the microRNA modulation represents an important mechanism through which various stimuli can rapidly modify important cellular processes, by targeting multiple genes at the same time [47].

Alterations of microRNA processing may also represent a way to perturbate cellular homeostasis. In fact, down-regulation of DICER, the RNAse III that together with the ribonuclease DROSHA are primarily implicated in the microRNAs processing, was shown to be a pro-tumorigenic event. This leads to enhance the epithelial to mesenchymal transition and the metastatic dissemination of breast cancer cells [48]. Moreover, lower expression of DICER is correlated with a worst prognosis of breast, lung and ovarian cancer patients [49,50].

The emerging role of microRNAs in lipid homeostasis has focused on finding possible strategies to block the activity of specific microRNAs by targeting with antisense oligos. The most known example is miR-122. It represents the most abundant microRNA in the liver and plays an important role in hepatic cholesterol and lipid metabolism (Figure 1). The antisense targeting of miR-122 into mice resulted in a decrease of plasma cholesterol levels [51,52] through the indirect modulation of genes involved in cholesterol biosynthesis, such as 3-hydroxy-3-methylglutaryl-CoA synthase 1 (Hmgcs1), 3-hydroxy-3-methylglutaryl-CoA reductase (Hmgcr) and 7-dehydrocholesterol reductase (Dhcr7) [52]. 

Similar to the miR-122 family of microRNAs, miR-33 is implicated as a potential target for metabolic disorders treatment. miR-33a and miR-33b are intronic microRNAs which are encoded together with their host genes, the sterol-regulatory element-binding proetein 1 (Srebp1) and 2 (Srebp2). The Srebp genes act coordinately with their intronic microRNAs to regulate cholesterol, triglyceride and lipid homeostasis (Figure 1).

In particular, Srebp2 and its intronic microRNA, miR-33a, regulate the cholesterol homeoastasis by modulating transcriptionally and post-transcriptionally the activity of genes involved in the cellular cholesterol export, such as the ATP-binding cassette (ABC) transporter ABCA1 and ABCG1 [53,54,55]. While, Srebp1 and miR-33b regulate the lipid homeostasis and the insulin signaling by modulating the activity of key genes involved in these process. However, there is not a specific separation of function between miR-33a and miR-33b. The two microRNAs shave overlapping gene targets through their similar mature sequence that differs of only two nucleotides. Several studies confirmed this evidence; thus, there is an extensive collaboration between miR-33a and miR-33b and their host genes (Figure 1).

miR-33-targeting antisense oligonucletotides appears to be a novel therapy approach for cardio metabolic disorders, such as atherosclerosis. The inhibition of ABCA1 by miR-33 induced an efflux of cholesterol from peripheral tissues to the liver and a consequent reduction of circulating high-density lipoprotein-cholesterol (HDL-C) [53]. In addition, the inhibition of the endogenous levels of miR33 in human liver cells induces fatty acid degradation, through the lack of modulation in the expression of genes involved in the oxidation of fatty acid [56,57]. Moreover, miR-33 have been involved in the post-transcriptional modulation of other mRNAs involved in the regulation of lipid and glucose metabolism, such as the α1 subunit of AMP-activated protein kinase (AMPKα1) [56,58,59]. The role of this enzyme in regulating energy homeostasis and tumor suppression will be discussed later in this review, as well as sirtuin 6 (Sirt6) [56,60].

However, Blandino and colleagues have shown in breast cancer cell lines a direct interaction between the miR-33a and the 3′-utr region of Myc mRNA. They suggested that the anticancer metabolic effects induced by metformin in breast cancer cell lines were, also a direct consequence of the Myc inhibition mediated by the up regulation of miR-33a. Metformin was not longer able to induce metabolic changes in breast cancer cell lines overexpressing c-Myc protein [61]. This evidence suggests that any anti-microRNA therapy must be designed for a specific tissue in a way to reduce the possible side effects.

The emerging role of altered metabolism in cancer development and progression makes it a target for a specific anticancer treatment. Metformin could be a candidate for metabolic targeted therapy. In fact, metformin is known to impinge on many metabolic pathways that result deregulated during cancer development. Indeed, its action modulates the activity of several key proteins whose anticancer roles will be extensive discussed in the following chapters.

## 3. Metformin Mechanism of Action

The mechanisms of metformin action are still being elucidated, however several studies underline its influence in the cellular energy balance. For example, multiple lines of evidence suggest that the action of metformin passes, largely, through the activation of the AMPK [62], although recent evidence has also suggested that the effects of this drug may be AMPK-independent [63]. With respect to the former hypothesis, metformin has been described to increase the cellular AMP:ATP ratio inhibiting the complex I of mitochondrial respiratory chain [10,64]. This change in AMP:ATP ratio and the consequence ATP depletion is sensed by AMPK, the master regulator of cellular energy homeostasis.

AMPK consists of three subunits, α, β and γ, and each subunit has at least two isoforms [65]. Activation of AMPK involves AMP binding to regulatory sites on the γ subunits. This causes conformational changes that allosterically activate the enzyme and inhibit dephosphorylation on Thr-172 within the activation loop of the catalytic α subunit [66,67]. The AMP binding on the γ subunit is not enough to activate AMPK. Liver kinase B1 (LKB1), a tumor suppressor gene, is required to phosphorylate the α subunit of AMPK at Thr-172 to activate the enzyme [68]. Once activated, AMPK regulates a pool of substrates which represents the key enzymes in the catabolic pathways and inhibits ATP-consuming anabolic pathways. AMPK also controls enzymes involved in cell cycle and protein metabolism.

Many studies indicate that loss of LKB1 impairs the ability of metformin to activate AMPK and maintain plasma glucose and insulin homeostasis (references). Moreover, patients affected by Polycystic Ovarian Syndrome (PCOS) showed a different ovulatory response once treated with metformin basing on their polymorphism in the LKB1 gene [69]. Hardie suggests that the LKB1 and AMPK pathway functions as a cellular energy-sensing checkpoint that controls cell growth and proliferation according to the availability of fuel supplies.

However, Foretz *et al.* [63] found that metformin inhibits liver glucose synthesis independently of LKB1 or AMPK status. In addition, Kalender *et al.* [70] demonstrated that metformin can enhance glucose uptake in rat skeletal muscle cells and inhibit mTOR signaling independently of AMPK. These findings suggest that a metformin AMPK-independent mechanism of action that interferes directly with the cellular energy output, and does not modulate genes involved in gluconeogenesis pathway, may exist [63]. Interestingly, the ataxia telangiectasia mutated (ATM) gene, a tumor suppressor gene involved in DNA repair and cell cycle control, was discovered to activate AMPK through LKB1 dependent and independent pathways [71,72].

## 4. The Pharmacokinetics of Metformin

Metformin entry into the cell via passive diffusion is limited due its hydrophilic nature. Thus, metformin requires the use of cation transporters, such as the organic cation transporters (OCTs), to facilitate its cellular uptake [73]. Currently, OCT1 and OCT2 which are encoded by the SLC22A1 and SLC22A2 genes, respectively, are recognized as the major transporters for metformin and are highly expressed in the liver, gut, and kidneys [74]. For example, mice that are null for OCT1 exhibit a significant reduction in hepatic metformin uptake, suggesting that the OCT1 transporter is essential for metformin accumulation in the liver, which is the main target tissue of this drug [75]. In addition, microarry analysis has revealed that the degree of OCT1 expression can be variable when comparing tumor *vs.* normal human tissues, suggesting that therapeutic responses to metformin in cancer patients may differ depending on their tumor-type [76].

By contrast, many *in vitro* models (experimental cell lines that are immortalized) have low expression of OCTs, and thus require millimolar (mM) concentrations of metformin to elicit similar physiological effect *in vivo* [73]. Although reports indicate that this issue can be bypassed by enhancing the ectopic expression of OCTs cDNAs into target cells [77,78]. Nevertheless, a recent report by Segal *et al.* [79] has indicated that OCT1 is expressed in multiple ovarian cancer cell lines, and that siRNA-mediated knockdown of OCT1 expression attenuated the ability of metformin (0–10 mM) to inhibit their survival.

Another important factor that may modulate the pharmacokinetics and therapeutic response to metformin are genetic variations in the OCT genes. To date, there have been at least 25 coding single nucleotide polymorphisms (cSNPs) identified in the SLC22A1 gene [80], many of which have been described to affect OCT1 function [81]. In addition, 14 polymorphisms in the SLC22A2 gene have been detected, and are implicated in regulating the renal clearance of metformin [82]. Such mutations may lead to complication with metformin toxicity, as 90% of metformin is cleared from the body within a few hours via the renal system.

Moving forward it will be important to better characterize other candidate genes that may influence metformin uptake treatment success. One genome-wide association study conducted in the United Kingdom, found that favorable glycemic responses to metformin were associated within a locus of the ATM gene [83]. This was an interesting finding since ATM is a DNA-damage sensor that is also implicated in regulating metformin-induced activation of AMPK in rat hepatocyte cells [83]. The effects of metformin on the ATM-AMPK cell cycle network may also explain some of chemopreventive proprieties (discussed in more detail below).

## 5. Metformin Anticancer Properties

The epidemiological evidences of the anticancer properties of metformin are supported by several molecular findings. Both AMPK-dependent and independent pathways have been proposed to mediate the anticancer effects of metformin treatment, which are described below.

### 5.1. AMPK Dependent Mechanisms

**Figure 2:**
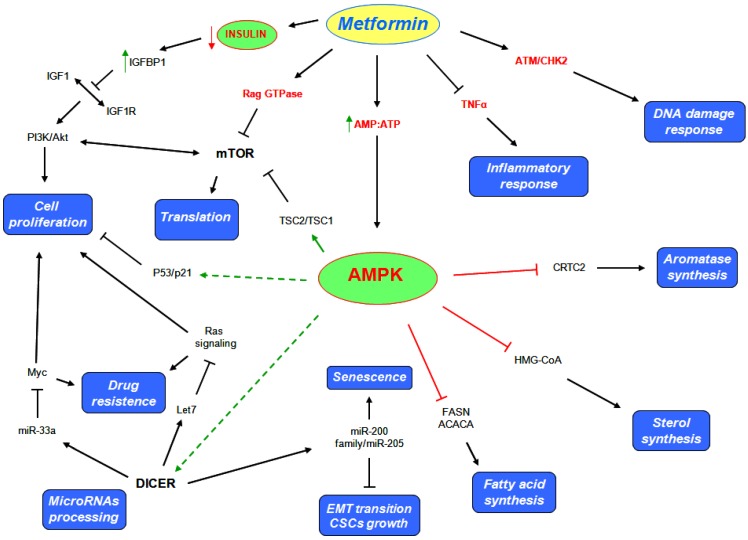
Metformin triggers multiple pathways to exert its anticancer activities.

As mentioned, AMPK is a key sensor of cellular energy stress. Metformin treatment induces a perturbation in the AMP:ATP ratio checked by AMPK, that once activated, phosphorylates substrates which promote the catabolic processes and inhibit the anabolic processes to restore energy homeostasis. The first substrate inhibited by AMPK play a key role in lipogenesis [23,83,84] (Figure 2). The inhibition of fatty acid synthesis (FAS), reduces the consumption of ATP and their consequence oxidation produces new ATP molecules, such to restore the ATP levels reduced by the metformin action. AMPK suppresses the activity of FASN and ACACA. In effect, hepatic cell cultures treated with metformin showed a decrease in ACACA and FASN levels [68], while no effects were observed in hepatic and skeletal muscle cells that lack of LKB1 [85]. In addition, high levels of FASN and ACACA were found in several pre malignant cells. Moreover, tumors such as breast, colon, prostate, and ovarian cancer, are characterized by a high rate of lipid metabolism. The *de novo* fatty acid synthesis is required for tumor formation and progression [28]. Not only lipid synthesis but also cholesterol synthesis play a pivotal role in the cancer cells metabolism. AMPK suppresses the activity of 3-hydroxy-3-methyl-glutaryl-CoA (HMG-CoA) reductase, thus to inhibit also cholesterol synthesis (Figure 2). Based on these evidences, metformin induces a shift in the lipid and cholesterol metabolism that could deprive pre-malignant and malignant cells of several substrates important for their growth and proliferation.

The PI3K/Akt/mTOR pathways are often deregulated in a large number of tumors. In particular, the anti mTOR therapies are an emerging strategy for renal cell carcinomas and breast cancer treatment. Several growth factors and nutrients stimuli impinge the PI3K/Akt/mTOR pathways, thus to sustain cell growth and proliferation [86]. mTORC1, one of the two functional mTOR complexes, regulates the protein synthesis by directly activating two important target involved in this process, such as S6 kinase (S6K) and translation initiation factor 4E binding protein 1 (4EBP1) [87]. AMPK negatively regulates mTORC1 by (i) modulating the activity of tuberous sclerosis complex 2 (TSC2), that together with TSC1 form a tumor suppressor complex that inhibits mTOR, or (ii) through phosphorylation and inhibition of its binding partner Raptor [88,89,90] (Figure 2). Moreover, metformin has been reported to inhibit the tumor progression of renal cells carcinoma by inhibiting mTOR activity through AMPK modulation [91]. Interestingly metformin exerts antiproliferative activity on glioblastoma cells through the inhibition of Akt pathway [92], besides, the inhibition of Akt activity seems to be the molecular mechanism by which metformin inhibits DNA damage accumulation in Drosophila midgut stem cell [93].

Furthermore, metformin represses aromatase expression in primary human breast adipose stromal cells, through the inhibition of CREB regulated transcription co-activator 2 (CRTC2) mediated by AMPK [94] (Figure 2). Based on this evidence, metformin treatment could represent a strategy for hormone-dependent post-menopausal breast carcinoma treatment, but it could be also a strategy for preventing post-menopausal breast carcinoma onset.

Activation of AMPK stimulates cell cycle arrest through p53/p21 axis [95] (Figure 2). Mutation of p53 on serine 18 impairs the ability of AMPK to induce cell cycle arrest [95]. In addition, the metabolic reprogramming induced by metformin seems to be p53 dependent. In effect, metformin selectively impairs p53-deficient tumor cells growth that unlike those carrying wild type p53 protein, are no able to reprogram their metabolism and become apoptotic [24]. Recently, metformin was reported to inhibit the proliferation of melanoma cell lines through the activation of the AMPK/p53 pathway [96].

Notably, metformin has been demonstrated to exert its anticancer action by increasing the DICER mRNA and protein levels in breast cancer cell lines (Figure 2). Metformin was no longer able to affect tumor engraftment in knock-down DICER cells [61]. As discussed, low levels of DICER were found in several tumors. Based on these evidences, the overexpression of DICER in cancer cell lines and the consequence change in their microRNAs profiling could be a possible anticancer mechanism of action mediated by metformin. Bao and colleagues confirmed the important role of metformin in microRNAs modulation. They indicated that pancreatic cell lines treated with metformin had re-expressed several microRNAs usually switched off in pancreatic cancer, such as let7, involved in regulation of Ras signaling [97], and the miR-200 family of microRNA, directly involved in epithelial to mesenchymal transition and in maintenance of the stem cell state [98]. In particular, they demonstrated that metformin inhibits tumor sphere formation by deregulating several cancer stem cell (CSC) markers (CD44, EpCAM, EZH2, Notch-1, Nanog and Oct4) in part through the up regulation of microRNAs [99]. Further evidences about metformin anticancer action and microRNAs modulation were also found in human pancreatic [100], esophageal squamous carcinoma [101], gastric [102] and prostate cancer cell lines [103].

Interestingly, metformin significantly increases the number of senescence-prone murine embryonic fibroblasts (MEFs) entering a senescent stage in response to DNA damages after doxorubicin treatment. This phenomenon is in part due to the modulation of the microRNAs 200 family and miR-205 [104] (Figure 2).

### 5.2. AMPK Independent Mechanisms

The main action of metformin is to sensitize peripheral tissue to insulin, thereby reducing insulin and glucose plasma levels. Interestingly, low circulating insulin levels induce the production of insulin like growth factor binding protein 1 (IGFBP1) in hyperinsulimenic women with PCOS syndrome [105] (Figure 2). PCOS patients show insulin resistance, hyperinsulinemia, anovulation and hyper-androgenemia. The latter aspect is promoted by a synergist action between luteinizing hormone (LH) and estrogen produced by granulose cells under insulin like growth factors 1 (IGF1) stimulation [106]. Based on this evidence, the increase in IGFBP1 levels mediated by metformin could limit the binding between IGF1 and their receptors (IGF1R) and consequently also the androgen production. Moreover, this metformin effect could synergize with the common anti androgen therapies, which are usually prescribed for metastatic prostate cancer treatment [107]. In addition, high levels of IGF-1 impair chemotherapy-induced apoptosis by activating the PI3K/Akt pathway (Figure 2). Inhibition of IGF1R sensitizes small cell lung cancer cell lines to the effects of etoposide and carboplatin combined treatment [108].

Quinn and colleagues found that metformin decreases lung tumorigenesis in liver IGF-I-deficient (LID) mice without affecting IGF-I levels. The authors suggested an IGF-I-independent mechanism of action exerted by metformin through the inhibition of circulating growth factors and local receptor tyrosine kinases (RTK) signaling in an AMPK independent way [109].

Metformin can also inhibit the PI3K/Akt/mTOR signaling pathway in an AMPK independent way by modulating Rag GTPase activity [70] (Figure 2) while, in lung tissue, metformin reduces the activity of mTOR by decreasing activation of IGFR1 and Akt [110]. Other several evidences show other possible metformin anticancer mechanisms of action. It was found to reduce chronic inflammatory response through the inhibition production of tumor necrosis factor alpha (TNFα) in human monocytes [111] (Figure 2). Moreover, it was demonstrated that metformin blocks the production of endogenous reactive oxygen species (ROS) by interfering the mitochondrial complex I activity [112]. In addition, metformin appears to sensitize cancer cells against further DNA damages through the modulation activity of checkpoint homolog kinase 2 (CHK2), which play an important role in mediating the DNA damage response of ATM [113,114] (Figure 2).

Interestingly, Ma *et al.* [115] demonstrated that cancer cell responsiveness to metformin is influenced by the K-ras status. Indeed, they showed that metformin induces apoptosis in the K‑ras mutant tumors, such as A549 and PANC‑1, but not in the K‑ras wild‑type tumor, A431, *in vitro*.

Recently, the Raf-ERK-Nrf2 axis and the subsequent down regulation of heme oxygenase-1 were proposed by Do and colleges to explain a new AMPK-independent metformin anticancer mechanism of action [116].

## 6. Metformin and Cancer Therapy

Metformin has also been studied for antineoplastic activity in patients with established cancer. The anticancer effects of metformin are via insulin-dependent and insulin-independent mechanisms.

Although metformin has become an attractive stand-all agent for molecular chemoprevention, many studies have also found it has a significant benefit when combined with common chemo or radiation therapy. For example, metformin has been shown to increase the cytotoxicity of many chemotherapeutic agents, including cisplatin, carboplatin, doxorubicin, and paclitaxel, and enhance tumor-free remission *in vivo* [117]. The ability of metformin to prolong tumor remission is believed to be due to the ability of this drug to selectively kill cancer stem cells (CSCs, also known as tumor initiating cells) that are chemoresistant and may lead to future cancer reoccurrence [118,119]. Indeed, metformin was found to eliminate CD44+/CD24− CSCs and sensitize in mouse breast cancer xenografts to doxorubicin treatment, leading to a significant reduction in the dose of chemotherapy used and thereby reducing complications due to toxicity [118]. In addition, metformin treatment was shown to reduced the proliferation rate of tumor-initiating cell-enriched cultures isolated from human glioblastomas [92].

Radiation therapy is another popular treatment modality for cancer of lung, breast, and prostate origin. However, many tumors are resistant to even high doses of ionizing radiation (IR), which sparked research interest into identifying adjuncts to radiotherapy [120]. Interestingly, Sanli *et al.* [120] identified that IR treatment activated the ATM-AMPK pathway in a variety of cancer cell lines, and that AMPK activity was required for IR-mediated G2/M cell cycle arrest. In addition, combination treatment with IR and metformin further potentiated AMPK signaling and sensitized lung cancer cells to the cytotoxic effects of IR. This same group later identified that clinically achievable doses of metformin (low μM range) were able to enhance radiation responses *in vivo* by blocking tumor angiogenesis and Akt-mTOR signaling, as well as enhancing apoptosis through the ATM-AMPK pathway [121].

In contrast to metformin, radiotherapy has also been found to increase the proportion of CSCs that survive IR treatment, indicating that CSCs are radio resistant compared to normal tumor cells. A recent report has indicated that metformin pretreatment in human MCF7 breast cancer or mouse FSaII fibrosarcoma cells can eradicate radiation-resistant CSCs and enhance IR-induced growth delay in fibrosarcoma tumors [122]. This recapitulated earlier reports that both IR and metformin activate AMPK and inhibit the mTOR pro-survival pathway.

There are several ongoing studies which look at the use of metformin in cancer therapeutics as documented by the National Cancer Institute (NCI). There are Phase I and II studies which look at the use of metformin in treatment of melanoma, chronic lymphocytic leukemia, glioblastoma multiforme, and cancers of the breast, pancreas, endometrium, and head and neck.

## 7. Role of Metformin as a Chemoprevention Agent

### 7.1. All Cancers

Observational data including registries of patients with type II diabetes have suggested that use of metformin may be associated with a decreased cancer incidence. Ruiter and colleagues published a large cohort study which included over 2.5 million diabetic patients who were identified via pharmacy dispensing records. They found that patients using metformin had a lower incidence of cancer compared to those who used sulfonylurea (HR 0.90, 95% CI 0.88–0.91) [123] (Table 1).

Currie *et al.* [124] published a retrospective cohort study in the UK, which included more than 100,000 patients from primary care practices, of which 8,392 had type II diabetes. All patients with or without diabetes who developed a tumor were followed for up to 19 years. Diabetes was stratified by treatment regimen and Cox proportional hazard models were used to compare all-case mortality from all cancers and from specific cancers. They found that cancer mortality increased in patients with diabetes compared to non-diabetic patients (HR 1.09, 95% CI 1.06–1.13). When analyzed according to type of cancer, mortality was increased in breast and prostate cancer but was decreased in lung cancer. However diabetic patients had a lower mortality rate than non-diabetic patients if they were treated with metformin (HR 0.85, 95% CI 0.78–0.93). Alternatively diabetic patients had a higher mortality than non-diabetic patients if they were treated with sulfonylurea (HR 1.13, 95% CI 1.05–1.21) (Table 1).

**Table 1:** Role of metformin in cancer therapy and chemoprevention.

Study	Study Types	Inclusion	Results
Franciosi, M 2013 *PLoS One*	Meta analysis 41 observational 12 RCT	Observational studies which examined association between metformin exposure and cancer. RCTs which compared metformin and other hypoglycaemic agents	Observational studies, metformin associated with: Decreased risk of death (6 studies, OR 0.65, 95% CI 0.53–0.80) Decreased all malignancies (18 studies, OR 0.73, 95% CI 0.61–0.88) Decreased liver cancer (8 studies, OR 0.34 95% CI 0.19–0.60) Decreased colorectal cancer (12 studies, OR 0.83, 95% CI 0.74–0.92) Decreased pancreas cancer (9 studies, OR 0.56, 95% CI 0.36–0.86) Decreased stomach cancer (2 studies, OR 0.83, 95% CI 0.76–0.91) Decrased esophageal cancer (2 studies OR 0.90 95% CI 0.83–0.98) In RCTs: No significant effect of metformin on death or malignancies
Thakkar, B 2013 *Metabolism*	Meta analysis 13 case contro l9 cohort 2 RCTs	Studies that assess metformin and/or sulfonylurea effects on cancer risk in diabetic patients	In observational studies, metformin use was associated with reduced risk of developing cancer: Cohort studies (RR 0.70, 95% CI 0.67–0.73) Case-control studies (OR 0.90 95% CI 0.84–0.98) In RCTs: No effect on developing cancer (RR 1.01 95% CI 0.81–1.26)
Stevens RJ 2012 *Diabetologia*	Meta analysis 13 RCTs	RCTs which compared metformin to another diabetic treatment or placebo/usual care, with minimum 500 participants and 1 year follow-up	In RCTs looking at cancer outcomes (11 studies): RR 1.02 (95% CI 0.82–1.26) (11 studies) In RCTs looking at all-cause mortality (13 studies): RR 0.94 (95% CI 0.79–1.12)
Soranna D., 2012 *Oncologist*	Meta analysis 17 observational	Observational studies of diabetic patients treated with metformin and/or sulfonylurea where risk of all cancers and specific cancer mortality was investigated	Metformin associated with decreased RR of: All cancers (RR 0.61, 95% CI 0.54–0.70) Colorectal cancer (RR 0.64, 95% CI 0.54–0.76) Pancreatic ca. (RR 0.38, 95% CI 0.14–0.91) Sulfonylurea not associated with reduced RR
Noto H., 2012 *PLoS ONE*	Meta analysis Cancer Mortality: 4 cohort 2 RCTs Cancer Incidence: 6 cohort 2 case control 2 RCTs	Studies of diabetic patients taking metformin compared to those not taking metformin	Metformin users compared to non users had: Lower cancer mortality (pooled RR 0.66, 95% CI 0.49–0.88) Lower cancer incidence (pooled RR 0.67, 95% CI 0.53–0.85) Lower colorectal ca. incidence (pooled RR 0.68, 95% CI 0.53–0.88) Lower HCC (pooled RR 0.20, 95% CI 0.07–0.59) Lower lung cancer (pooled RR 0.67, 95% CI 0.45–0.99)

Home *et al.* [125] looked at the outcomes of patients enrolled in two specific randomized controlled trials, ADOPT (A Diabetes Outcome Progression Trial) and RECORD (Rosiglitazone Evaluated for Cardiovascular Outcomes and Regulation of Glycaemia in Diabetes), which tested the safety and efficacy of metformin in comparison to sulfonylureas and rosiglitazone. These studies did not show statistically significant differences in cancer outcomes for patients treated with metformin. In ADOPT the hazard ratio for developing a serious adverse event malignancy (excluding non-melanoma skin cancers) was 0.92 (95% CI 0.63–1.35) when compared to rosiglitazone and was 0.78 (95% CI 0.53–1.14) when compared to glibencalmide. In RECORD, on a background of sulfonylurea, the hazard ratio of developing malignant neoplasm in metformin *versus* rosiglitazone group was 1.22 (95% CI 0.86–1.74). Although these studies suggest a trend for an association between metformin and risk of malignancy, there was no statistically significant association (Table 1).

Five recent meta-analysis have pooled data from studies which look at the potential use of metformin in cancer prevention in diabetic patients [126,127,128,129,130]. The largest and most recent meta analysis included 12 randomized controlled trials which compared metformin and other diabetic medications, and 41 observational studies which examined the association between metformin and cancer. In randomized controlled trials, there was no significant association between metformin exposure and risk of cancer death or of malignancy [126]. The observational studies found a significant association between metformin exposure and risk of cancer death, OR 0.65 (95% CI 0.53–0.80) and all malignancies, OR 0.73 (95% CI 0.61–0.88).

Similar to this study, another meta analysis included 24 studies which examined subjects with type II diabetes and the effects of metformin and/or sulfonylurea on risk of cancer [127]. They found that metformin was associated with reduced risk of cancer in both cohort (RR = 0.70, 95% CI 0.67–0.73) and case control (OR = 0.90, 95% CI 0.84–0.98) studies, but not in RCTs (RR = 1.01 95% CI 0.81–1.26). In the same study, data from 18 studies which examined the effects of sulfonylurea in cancer prevention found an increase in all-cancer risk in cohort studies (RR = 1.55, 95% CI 1.48–1.63), but there was no statistically significant effect in case-control or RCT studies.

One meta analysis included only randomized controlled trials [128]. Results did not show a statistically significant benefit of metformin on cancer outcomes including incidence and mortality. In 11 randomized controlled trials which looked at cancer outcomes, the relative risk in patients randomized to metformin compared to a comparator was 1.02 (95% CI 0.82–1.26). In 13 randomized controlled trials which looked at all-cause mortality, the relative risk was 0.94 (95% CI 0.79–1.12). The authors identified limitations in the trials including heterogeneous comparator groups and short follow-up. Follow up varied in each study, ranging from 1 year to 10.7 years, and the results were reported according to 51,681 person-years. Another very important limitation is that none of the clinical trials were designed to look at cancer as the primary outcome.

One meta-analysis included only prospective studies which investigated the risk of all cancers and specific cancer sites in patients with type II diabetes who used metformin and/or sulfonylurea [129]. In 17 studies, the use of metformin was associated with a decreased relative risk of all cancer (RR 0.61, 95% CI 0.54–0.70). When subdivided by type of cancer, the relative risk for colon cancer was 0.64 (95% CI 0.54–0.76); for pancreatic cancer was RR = 0.38 (95% CI 0.14–0.91). There was no evidence of association between metformin and reduction of breast and prostate cancers. There was no decrease in the relative risk of all or any cancer in patients who were treated with sulfonylurea. Using Egger’s regression asymmetry test and funnel plot, the authors found that evidence for publication bias for metformin-cancer association was present. This brings about a very important point. Publication bias is a factor which must be taken into consideration when interpreting the results of any meta analysis.

The first meta analysis to suggest a survival benefit with metformin use in diabetic patients included four cohort studies plus two randomized controlled trials (RR 0.66, 95% CI 0.49–0.88) [130]. Metformin users also had lower cancer incidence (pooled RR 0.67, 95% CI 0.53–0.85). When subdivided by cancer type, there was lower incidence of colorectal cancer (pooled RR 0.68, 95% 0.53–0.88), hepatocellular carcinoma (pooled RR 0.20, 95% CI 0.07–0.59), and lung cancer (pooled RR 0.67, 95% CI 0.45–0.99). However, there were no statistically significant benefits of metformin in reducing incidence of breast, prostate, pancreatic, gastric, or bladder cancer.

The association of cancer outcomes and mortality with metformin use has been found in observational and cohort studies. These studies, however, inherently have biases which can be addressed with the use of randomized controlled trials. For example, there is evidence that a majority of observational studies which look at metformin and cancer suffer from immortal time bias and time-window bias which can lead to an exaggerated treatment effect [131]. To date, as outlined above, there are no meta analysis of randomized controlled trials which have shown a statistically significant effect of metformin on cancer outcomes. However the randomized clinical trials which are included in these analyses do not examine cancer as the primary outcome. There is a need for randomized controlled trials which compare individual treated with metformin compared to those who are not, and which look specifically for cancer outcomes.

### 7.2. Colorectal Cancer

A meta-analysis was published in 2011 which included 4 studies with more than 100,000 patients with type II diabetes who were treated with metformin and in whom colorectal cancer outcomes were reported [132]. A random-effects meta-analysis model was used to summarize their findings. Metformin treatment was associated with a significantly lower risk of colorectal cancer with RR = 0.63 (95% CI 0.47–0.84). The authors concluded that further clinical trials are required to verify these results which suggest an association between metformin use and decreased colorectal risk (Table 1).

### 7.3. Lung Cancer

A case control study in Taiwan looked at National Health Insurance claims compared 19,624 cases of newly diagnosed diabetes patients with 78,496 controls who were matched in sex and age to cases and they were followed for 9 years [133]. A multivariate Cox model analysis did not show a significant increase in lung cancer in diabetes patients compared to controls (HR = 1.05). In patients who were treated with anti-diabetic medication, there was a 39%–45% decreased risk of lung cancer. This included patients treated with metformin, thiazolidinediones, or alpha-glucosidase inhibitors (Table 1).

### 7.4. Hepatocellular Carcinoma

A meta analysis which included 5 studies and 105,495 patients with type II diabetes was published by Zhang and colleagues [134]. The effects of metformin on risk of liver cancer were analyzed with a random-effects model. The risk of liver cancer risk was decreased by 62% in diabetic patients who were treated with metformin (OR 0.38, 95% CI 0.24–0.59). The risk of hepatocellular carcinoma in particular was assessed in 4 trials and was decreased by 70% in diabetic patients who were treated with metformin (OR 0.30, 95% CI 0.17–0.52) (Table 1).

### 7.5. Breast Cancer

Prevention of breast cancer incidence in diabetic patients on metformin has been studied in the large Women’s Health Initiative cohort study [135]. In this study, 3401 women with type II diabetes were compared for breast cancer incidence to women without diabetes. Diabetic women did not have a significantly higher incidence of breast cancer compared to non-diabetic women (HR 1.16, 95% CI 0.93–1.45). Interestingly, diabetic women treated with metformin had a lower incidence of breast cancer when compared to non-diabetic women who were not on metformin (HR 0.75, 95% CI 0.57–0.99). A nested case control study was conducted in Denmark which included 393 diabetic patients and 3930 controls [136]. Patients on metformin were less likely to have a breast cancer diagnosis (OR 0.77, 95% CI 0.61–0.99) (Table 1).

### 7.6. Ongoing Studies

There are several ongoing studies which are looking to determine the role of metformin in cancer prevention. Currently there are 168 studies registered worldwide on clinicaltrials.gov. Out of these, 77 are registered in North America, 52 in Europe, 14 in Asia, 8 in Africa, and 6 in South America. Specifically by cancer site, there are 24 studies in breast cancer, 11 studies in prostate cancer, 7 studies in colorectal cancer and 4 studies in lung cancer along with many others. These studies include phase I, phase II, and phase III studies.

## 8. Conclusions

Conclusive evidence has shown that metabolic alteration is a key future of cancer cells. Furthermore, metabolic alteration is a multistep process which occurs through the spatial and temporal disabling of specific biochemical pathways. This is early driven by either loss of tumor suppressor or aberrant oncogenic activities which lead to altered metabolism.

The anticancer activity of metformin which causes metabolic reprogramming of cancer cells through the modulation of non-coding RNAs, is filling the gap of mechanistic knowledge of the epidemiologic evidence showing that diabetic patients treated with metformin have reduced cancer incidence and cancer-related mortality. This is also well documented by the large number of ongoing clinical trials using metformin in cancer treatment. Additionally, this growing mechanistic evidence is providing a strong rationale for the implementation of chemoprevention trials whose success could have an enormous impact on the health worldwide.
